# The Impact of Acute Ammonia Nitrogen Stress on Serum Biochemical Indicators and Spleen Gene Expression in Juvenile Yellowfin Tuna (*Thunnus albacares*)

**DOI:** 10.3390/ani14213090

**Published:** 2024-10-26

**Authors:** Yongyue Sun, Zhengyi Fu, Zhenhua Ma

**Affiliations:** 1 Key Laboratory of Efficient Utilization and Processing of Marine Fishery Resources of Hainan Province, Sanya Tropical Fisheries Research Institute, Sanya 572018, China; 2 South China Sea Fisheries Research Institute, Chinese Academy of Fishery Sciences, Guangzhou 510300, China; 3 Hainan Engineering Research Center for Deep-Sea Aquaculture and Processing, Sanya Tropical Fisheries Research Institute, Sanya 572018, China; 4 International Joint Research Center for Conservation and Application of Fishery Resources in the South China Sea, Sanya Tropical Fisheries Research Institute, Sanya 572018, China; 5 College of Fisheries and Life Science, Shanghai Ocean University, Shanghai 201306, China; 6 College of Science and Engineering, Flinders University, Adelaide 5001, Australia

**Keywords:** serum biochemistry, gene mRNA expression levels, immunobiochemical indicators, pro-inflammatory, apoptosis

## Abstract

The presence of ammonia nitrogen in water has a significant impact on the serum and spleen of fish, potentially leading to changes in substances such as proteins in the serum while also causing damage to the immune function of the spleen. To investigate the effects of ammonia nitrogen (NH_3_-N) stress on juvenile yellowfin tuna (*Thunnus albacares*), this study established three NH_3_-N concentrations, 0, 5, and 10 mg/L, denoted as L0, L1, and L2, respectively. Serum and spleen samples were collected at 6, 24, and 36 h. The effects of different NH_3_-N concentrations and exposure times on the physiological status of juvenile fish were investigated by analyzing serum biochemical indices and splenic gene expression. The results indicate that in the L1 group, the serum high-density lipoprotein cholesterol (HDL-C), triglycerides (TG), complement 3 (C3) and complement 4 (C4) levels, and acid phosphatase (ACP) activity showed a trend of initially increasing and then decreasing. In the L2 group, the serum low-density lipoprotein cholesterol (LDL-C), HDL-C, and C4 levels and ACP activity also displayed an initially rising and then declining trend, while TG, C3, and creatinine (CRE) levels and alkaline phosphatase (AKP) activity showed an upward trend. In the L1 group, glutathione peroxidase 1b (*GPX1b*), interleukin 10 (*IL-10*), interleukin 6 receptor (*IL-6r*), and tumor necrosis factor alpha (*TNF-α*) gene expression levels in the spleen exhibited a trend of first increasing and then decreasing. In the L2 group, *IL-10*, *IL-6r*, tumor necrosis factor beta (*TNF-β*), caspase 2 (*casp2*), and caspase 9 (*casp9*) gene expression levels in the spleen also showed an initial increase followed by a decrease. When NH_3_-N levels are below 5 mg/L, it is recommended to limit stress exposure to no more than 36 h for the juvenile fish. For concentrations ranging from 5 to 10 mg/L, stress should be strictly controlled to within 24 h. Exposure to high NH_3_-N levels may affect biochemical indicators such as serum lipid metabolism, immunity, and metabolism in juvenile fish, and may damage the expression of antioxidant, immune gene, and apoptosis factors in the spleen. This study aims to deepen our understanding of the effects of NH_3_-N on juvenile tuna, with the goal of establishing effective water quality monitoring and management strategies. This will ensure the quality of aquaculture water, reduce the harm caused by NH_3_-N to juvenile yellowfin tuna, and enhance aquaculture efficiency and product quality.

## Simple Summary

To better understand the changes in serum biochemical indicators and spleen gene expression in yellowfin tuna (*Thunnus albacares*), this study aims to provide data support for common issues encountered during the aquaculture of yellowfin tuna. The components in the serum of yellowfin tuna are involved in various physiological processes, while the spleen plays a crucial role in the immune system. However, there has been limited research into the biochemical analysis of serum and spleen gene expression in farmed yellowfin tuna in China. In this study, natural seawater was used as a control group, with ammonia nitrogen concentrations set at 5 mg/L and 10 mg/L. The fish were treated for 36 h under varying ammonia nitrogen concentrations, and serum biochemical indicators and spleen gene expression of juvenile yellowfin tuna were measured at different time points (6 h, 24 h, and 36 h) to provide data support for the aquaculture of juvenile yellowfin tuna.

## 1. Introduction

Ammonia nitrogen (NH_3_-N) is the end product of protein catabolism and the most common water environmental limiting factor in aquaculture [1]. The primary sources of increased NH_3_-N concentration in aquaculture waters include the decomposition of nitrogenous organic matter, such as feed residues and excreta from aquatic animals, as well as the discharge of industrial and domestic wastewater [2,3]. NH_3_-N can affect enzyme metabolism, causing metabolic disorders in enzymes, reducing immunity, and triggering a series of toxic reactions in fish, such as excitement and convulsions, leading to exhaustion and death. Research indicates that NH3-N can alter serum biochemical indicators, antioxidant capacity, immune substances, and inflammatory factors in species such as hybrid grouper (♀ *Epinephelus fuscoguttatus* × ♂ *E. lanceolatu*), juvenile yellowfin tuna (*Thunnus albacares*), and barramundi (*Lates calcarifer*) [4,5,6].

Serum biochemical indicators serve as a crucial means to assess the physiological state and health condition of aquatic organisms [7,8]. High-density lipoprotein cholesterol (HDL-C), low-density lipoprotein cholesterol (LDL-C), total cholesterol (T-CHO), and triglycerides (TG) in serum are all vital indicators for evaluating lipid metabolism levels in the bloodstream [9,10,11,12,13]. HDL-C possesses immunological functions, whereas LDL-C can induce vascular inflammation [12,13]. Research indicates that under NH_3_-N stress, the serum CHO concentration in the half-smooth tongue sole (*Cynoglossus semilaevis*) decreases, while the levels of HDL-C and LDL-C remain unchanged [14]. Complement is a vital component of the immune system, playing a significant role in the body’s defense against pathogenic bacteria and inflammatory responses [15]. Acid phosphatase (ACP) and alkaline phosphatase (AKP) play crucial roles in the digestive, absorptive, and transport mechanisms of specific nutrients, serving as integral components of the organism’s detoxification pathways [16]. Urea nitrogen (BUN) and creatinine (CRE) are non-protein nitrogenous waste (NPN), and their levels inversely reflect the glomerular filtration function of the kidneys [17,18]. Current research has investigated the changes in BUN and CRE levels in the serum of aquatic animals such as the juvenile Japanese pufferfish (*Takifugu rubripes*) and juvenile cobia (*Rachycentron canadum*) [19,20].

NH_3_-N stress can lead to various “stresses” that fish cells must repeatedly confront, requiring the genome to respond in a programmed manner for the organism to survive. For instance, in response to oxidative stress, antioxidant genes are activated [21]. Superoxide dismutase (SOD), catalase (CAT), and glutathione peroxidase (GSH-PX) are essential components of the antioxidant defense system [22]. Research indicates that hybrid groupers upregulate antioxidant genes in response to NH_3_-N stress [23]. Additionally, NH_3_-N stress can trigger inflammatory responses in fish. An overactive inflammatory response can lead to cell necrosis or apoptosis due to the excessive activity of pro-inflammatory cytokines. To balance the pro-inflammatory cascade reaction, the body releases anti-inflammatory cytokines. These anti-inflammatory mediators suppress immune cells and lead to immunosuppression [24,25,26]. Common pro-inflammatory factors include interleukin 6 (IL-6), tumor necrosis factor a (*TNF-α*), and tumor necrosis factor b (*TNF-β*), while common anti-inflammatory factors include interleukin 10 (*IL-10*) [24,27]. Studies have found that NH_3_-N stress induces inflammatory responses in the redfin pufferfish (*Takifugu rubripes*) and Japanese sea bass (*Lateolabrax japonicus*), leading to changes in immune gene expression [28,29]. NH_3_-N stress not only causes oxidative stress and immune responses in fish but also activates apoptosis. Both caspase 2 (*casp2*) and caspase 9 (*casp9*) are initiators of the intrinsic pathway of apoptosis [30,31]. Recent research has investigated caspase genes in organisms such as the Pacific cod (*Gadus*), orange-spotted grouper (*Epinephelus coioides*), and gilthead seabream (*Sparus aurata*) [32,33,34].

The yellowfin tuna is a species of bony fish that migrates between the northern and southern waters of tropical and subtropical regions, and which belongs to the family Scombridae within the order Perciformes [35]. Rich in polyunsaturated fatty acids, yellowfin tuna is crucial for human growth and development [36,37,38]. Currently, there is an urgent need for artificial breeding of yellowfin tuna to address the issue of overfishing [39]. As a key supplier of yellowfin tuna, China has developed indoor recirculating aquaculture and offshore deep-water cage farming techniques for this species at the Tropical Aquatic Research and Development Center (TARDC) in Xincun Town, Lingshui, Hainan Province. Research on the nutritional evaluation, reproductive performance, fishery resources, and biochemical indicators of yellowfin tuna has been conducted, but the scarcity of publicly available breeding data has limited the development of artificial breeding for this species [40,41,42,43].

As awareness of marine conservation grows, an increasing number of researchers are studying harmonious coexistence with marine animals. However, systematic research into the specific species of yellowfin tuna remains insufficient. This study explores the effects of acute NH_3_-N stress on the serum biochemical indicators and related spleen genes in juvenile yellowfin tuna. This research will contribute valuable scientific evidence for marine environmental management, offer strategic recommendations for the sustainable utilization of fishery resources, and provide new insights into the environmental adaptability of marine organisms. This study investigates the changes in serum biochemical indicators and spleen gene expression of juvenile tuna during their growth in relation to ammonia nitrogen concentration. It aims to explore the adaptability of serum biochemical indicators and spleen genes to ammonia nitrogen levels, contributing experimental data and research materials for the aquaculture, ecological physiology, and domestication of juvenile yellowfin tuna.

## 2. Materials and Methods

### 2.1. Experimental Fish and Design

The experiment was conducted at the TARDC. The experimental subjects were juvenile yellowfin tuna, sourced from aquaculture cages in the adjacent sea area. The average body length was 22.33 ± 2.28 cm, and the average weight was 260.39 ± 55.99 g. During the adaptation period, the water temperature was maintained at 29 ± 1 °C, the salinity was 32‰, the oxygen level was kept at 7.00 ± 0.50 mg/L, and the concentrations of NH_3_-N and nitrite were controlled below 0.01 mg/L. Prior to the start of the experiment, juvenile fish were adapted for one week in a recirculating aquaculture system.

According to the methods outlined in the literature, the ammonia nitrogen concentrations in the preliminary experiment were set to 0 (natural seawater, control group), 5, 10, and 20 mg/L [6,44,45]. All the fish in the 20 mg/L group died within 2 h. For the formal experiment, the ammonia nitrogen concentrations were set to 0 (natural seawater, control group), 5, and 10 mg/L, labeled as L0, L1, and L2, respectively. Based on the dynamic equilibrium formula for NH_3_ and NH_4_^+^, the NH_3_ concentrations for the three groups were calculated to be 0 mg/L, 0.45 mg/L, and 0.91 mg/L, respectively [46]. Each concentration was tested three times, with each trial consisting of 20 fish, totaling 180 fish. A 10 g/L stock solution of ammonium chloride (NH_4_Cl) with 99.5% purity (Xi Long Chemical Co., Ltd., Foshan, China) was prepared as the source of ammonia nitrogen. A portable water quality detector (Aokedian Biotechnology Co., Ltd., Wuxi, China) was employed to monitor NH_3_-N concentrations, with measurements taken every two hours. No feeding was conducted 24 h prior to or during the experiment.

At 6, 24, and 36 h, three yellowfin tuna were randomly selected from each replicate and these nine fish were anesthetized using MS 222 (Tricaine methanesulfonate, Merck & Co., Inc., Rahway, NJ, USA). MS 222 was added to the water slowly, according to the instructions, until the juvenile yellowfin tuna stopped swimming. Using a disposable syringe (pre-rinsed with 1% sodium heparin), blood was drawn from the tail veins of these nine juvenile tuna into 2 mL centrifuge tubes. The samples were immediately centrifuged (Heraeus Corporation, Hanau, Germany, 3500 rpm, 10 min, 4 °C), and the supernatants were collected in 2 mL centrifuge tubes and stored at −80 °C for subsequent analysis. Simultaneously, these nine juvenile tuna were dissected on ice to collect the spleens, which were placed in cryotubes and then transferred to a liquid nitrogen tank. The next day, the samples were taken out and stored in a −80 °C freezer for subsequent analysis and measurement. The determination of serum biochemical indicators and spleen gene expression was completed within one month.

### 2.2. Institutional Review Board Statement

The experiment complied with the regulations and guidelines established by the Animal Care and Use Committee of the South China Sea Fisheries Research Institute, Chinese Academy of Fishery Sciences. The approval number is 2020TD55, and it was approved on 5 January 2020.

### 2.3. Determination of Serum Biochemical Parameters

First, serum biochemical parameters were measured using a biochemical test kit (Jiancheng Bioengineering Institute, Nanjing, China). Subsequently, absorbance values were recorded with a microplate reader (BioTek Instruments, Inc., Winooski, VT, USA). The HDL-C and LDL-C levels were determined using enzymatic modification methods with the HDL-C test kit (Catalog No. A112-1-1) and LDL-C test kit (Catalog No. A113-1-1), respectively, measuring their absorbance at 600 nm. The TG concentration was measured using the TG test kit (Catalog No. A110-1-1) with the glycerophosphate oxidase-peroxidase assay procedure, which utilizes the specific enzymatic reaction to quantify TG. The T-CHO concentration was determined using the cholesterol oxidase–peroxidase assay procedure with the T-CHO test kit (Catalog No. A111-1-1). Complement 3 (C3) (Catalog No. E032-1-1) and complement 4 (C4) (Catalog No. E033-1-1) were measured using immunoturbidimetry, where the C3 antibody reacts with the antigen to form immune complexes. The turbidity changes were detected at 340 nm, with the variation directly proportional to the C3 concentration; the same principle applies to C4. The ACP and AKP levels were determined using the ACP test kit (Catalog No. A060-2) and AKP test kit (Catalog No. A059-2), respectively. In alkaline solution, they first react with 4-aminoantipyrine, then are oxidized by potassium ferricyanide to form quinone derivatives, which are red in color. The enzyme activity is determined by the depth of the red color. The BUN concentration was measured using the BUN test kit (Catalog No. C013-2-1) with the urease method, where urea is hydrolyzed by urease to produce ammonia ions. In alkaline conditions, ammonia reacts with a phenol chromogenic reagent to form a blue substance, which is measured at 640 nm. The amount of this substance is directly proportional to the BUN concentration. The CRE concentration was determined using the CRE test kit (Catalog No. C011-2-1) with the sarcosine oxidase method at 546 nm.

### 2.4. Spleen RNA Extraction and Reverse Transcription

First, RNA was extracted from the spleen using Trizol reagent (Labgic Technology Co., Ltd., Hefei, China). The 260/280 nm absorbance ratio was used as an indicator of RNA quality. Subsequently, RNA purity was assessed using a spectrophotometer (Nano-300, Hangzhou Aosheng Instruments Co., Ltd., Hangzhou, China). An ideal ratio falls within the range of 1.8 to 2.0, signifying high purity and integrity. The quality of the RNA was further evaluated through denaturing agarose gel electrophoresis (1% TAE) (Labgic Technology Co., Ltd., Hefei, China). The synthesis of cDNA was performed using the SuperMix kit (Jinshi Biotechnology Co., Ltd., Hangzhou, China) for reverse transcription.

### 2.5. Gene Expression Analysis

Primer design was conducted using Primer Premier 5 software (Premier Biosoft, Waterloo, ON, Canada), with the primer sequences detailed in Table 1. The primers were synthesized by Sangon Biotech (Sangon Biotech (Shanghai) Co., Ltd., Shanghai, China). The reference gene used was β-actin [47], and the gene sequences were obtained from Shanghai Majorbio Bio-pharm Technology Co., Ltd. (Shanghai, China).

In this study, SYBR green fluorescent quantitative PCR kits (TianGen Biotechnology Co., Ltd., Beijing, China) and real-time quantitative PCR (qRT-PCR) instruments (Langji Scientific Instruments Co., Ltd., Hangzhou, China) were used. The specific amplification protocol was as follows: an initial denaturation at 95 °C for 15 min, followed by 40 cycles of denaturation at 95 °C for 10 s, annealing at 60 °C for 20 s, and extension at 72 °C for 30 s [47,48]. All PCR amplification reactions were performed in a 20 μL reaction mixture, consisting of 10 μL 2× RealUniversal PreMix, 0.6 μL of each forward and reverse primer (10 μM), 6.8 μL RNase-free ddH_2_O, and 2 μL cDNA template [47,48]. Data analysis was conducted using the 2^−ΔΔCt^ method.

### 2.6. Statistical Analysis

In this experiment, data are represented using the mean ± standard deviation (mean ± SD). SPSS 25.0 software (IBM Corporation, Armonk, NY, USA) was employed for one-way analysis of variance (one-way ANOVA) to analyze the data. Prior to analysis, normality and homogeneity of variance tests were conducted on the data. If significant differences were observed (*p* < 0.05), the Duncan multiple range test was performed to compare differences between groups. Origin 2022 software (OriginLab Corporation, Northampton, MA, USA) was utilized to graphically represent the experimental data.

## 3. Results

### 3.1. The Effects on Serum Lipid Metabolism Biochemical Indicators

**Figure 1:**
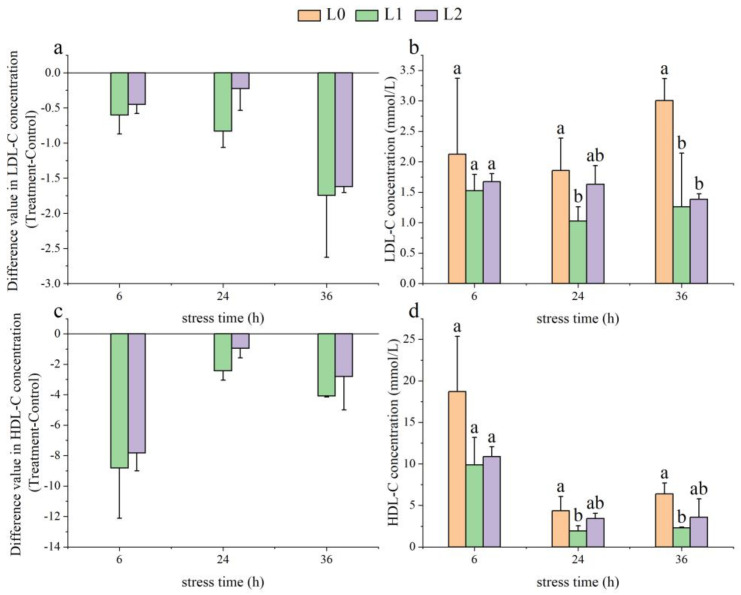

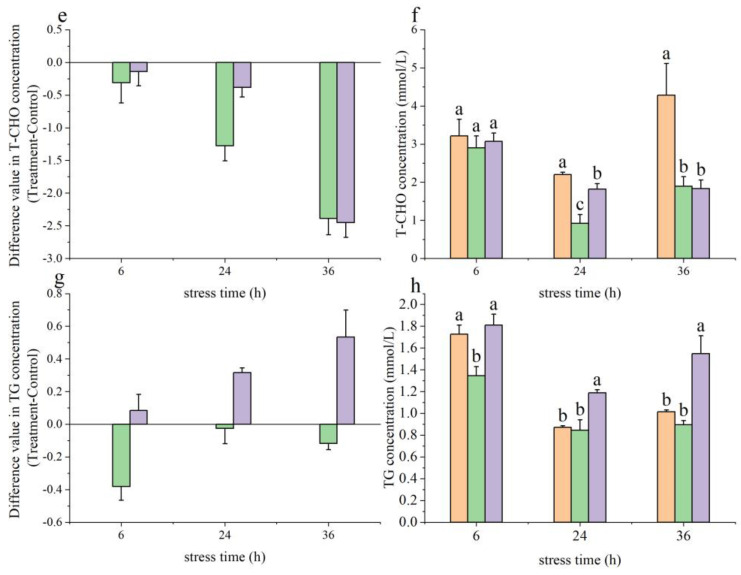
Effects of acute ammonia nitrogen stress on biochemical indices of serum lipid metabolism in juvenile yellowfin tuna (*Thunnus albacares*) (n = 9). (**a**) Difference value in low-density lipoprotein cholesterol (LDL-C) concentration (treatment − control), (**b**) LDL-C concentration in treatment and control groups, (**c**) difference value in low-density lipoprotein cholesterol (HDL-C) concentration (treatment − control), (**d**) HDL-C concentration in treatment and control groups, (**e**) difference value in total cholesterol (T-CHO) concentration (treatment − control), (**f**) T-CHO concentration in treatment and control groups, (**g**) difference value in triglyceride (TG) concentration (treatment − control), and (**h**) TG concentration in treatment and control groups. The difference value = treatment value − control mean value. Different letters indicate statistically significant differences between ammonia concentrations at the same time point (*p* < 0.05).

As time went on, the LDL-C activity in the L1 group showed a declining trend, decreasing by −1.74 ± 0.88 mmol/L at 36 h. In contrast, the LDL-C levels in the L2 group initially rose before declining, with a decrease of −1.62 ± 0.09 mmol/L at 36 h (Figure 1a). At 24 and 36 h, the LDL-C levels in the L1 group were significantly lower than those in the L0 group (*p* < 0.05). Additionally, at 36 h, the LDL-C levels in the L2 group were also significantly lower than those in the L0 group (*p* < 0.05, Figure 1b).

As time progressed, the HDL-C activity in the L1 and L2 groups showed an initial increase followed by a decline, with decreases of −8.82 ± 3.29 mmol/L and −7.83 ± 1.18 mmol/L at 6 h, respectively (Figure 1c). At 24 and 36 h, HDL-C levels were significantly lower than those in the L0 group (*p* < 0.05), while the HDL-C levels in the L2 group showed no significant difference compared to the L0 group (*p* > 0.05, Figure 1d).

As time progressed, the T-CHO levels in the L1 and L2 groups showed a decreasing trend, dropping by −2.39 ± 0.25 mmol/L and −2.45 ± 0.23 mmol/L at 36 h, respectively (Figure 1e). At 24 and 36 h, the T-CHO levels in the L1 and L2 groups were significantly lower than those in the L0 group (*p* < 0.05) (Figure 1f).

With increasing time, the TG levels in the L1 group first rose and then fell, decreasing by −0.38 ± 0.08 mmol/L at 6 h. In contrast, the TG levels in the L2 group showed an upward trend, increasing by 0.53 ± 0.17 mmol/L at 36 h (Figure 1g). At 6 h, the TG levels in the L1 group were lower than those in the L0 group (*p* < 0.05), while at 24 and 36 h, the TG levels in the L2 group were significantly higher than those in the L0 group (*p* < 0.05, Figure 1h).

### 3.2. The Effects on Serum Immunobiochemical Indicators

**Figure 2:**
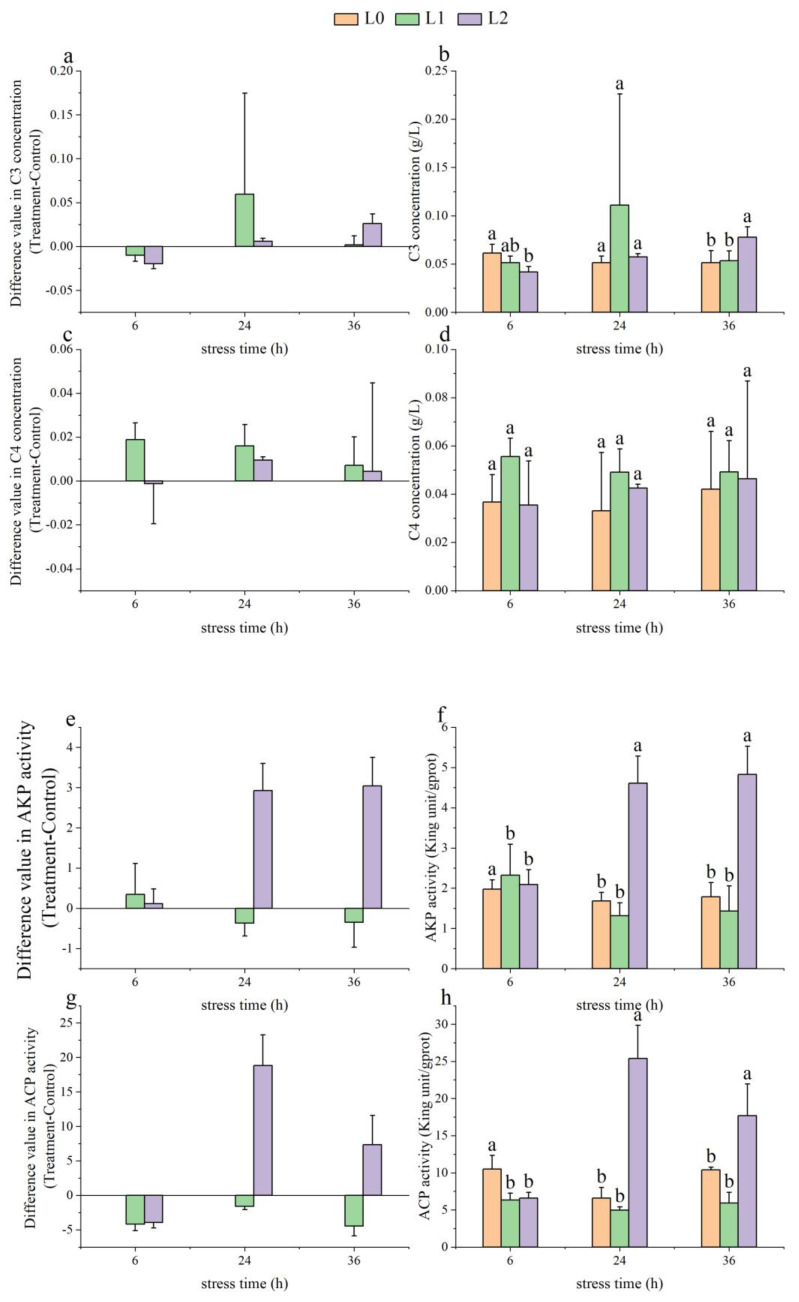
Effect of acute ammonia nitrogen stress on immuno-biochemical indices in the serum of juvenile yellowfin tuna (*Thunnus albacares*) (n = 9). (**a**) Difference value in Complement 3 (C3) concentration (treatment − control), (**b**) C3 concentration in treatment and control groups, (**c**) difference value in Complement 4 (C4) concentration (treatment − control), (**d**) C4 concentration in treatment and control groups, (**e**) difference value in alkaline phosphatase (AKP) activity (treatment − control), (**f**) AKP activity in treatment and control groups, (**g**) difference value in acid phosphatase (ACP) activity (treatment − control), and (**h**) ACP activity in treatment and control groups. The difference value = treatment value − control mean value. Different letters denote statistically significant differences (*p* < 0.05) between ammonia concentrations at the same time point.

As time increased, the C3 levels in the L1 group showed a trend of initially rising and then falling, with an increase of 0.06 ± 0.12 g/L at 24 h. The C3 levels in the L2 group showed an upward trend, increasing by 0.03 ± 0.01 g/L at 36 h (Figure 2a). There was no significant difference in C3 levels between the L1 and L0 groups (*p* > 0.05), while the C3 levels in the L2 group were significantly lower than L0 at 6 h (*p* < 0.05) and significantly higher than L0 at 36 h (*p* < 0.05, Figure 2b).

With increasing time, the C4 levels in the L1 group showed a downward trend, increasing by 0.02 ± 0.01 g/L at 6 h. The C4 levels in the L2 group exhibited an initial rise followed by a decline, increasing by 0.01 ± 0.01 g/L at 24 h (Figure 2c), with no significant differences among the three groups from 6 to 36 h (*p* > 0.05).

The AKP activity in the L1 group decreased from 6 to 24 h, increasing by 0.35 ± 0.77 King unit/gprot at 6 h, while the AKP activity in the L2 group increased during this period, rising by 2.93 ± 0.67 King unit/gprot at 24 h (Figure 2e). At 24 and 36 h, the AKP activity in the L2 group was significantly higher than in the L0 and L1 groups (*p* < 0.05, Figure 2f).

As time progressed, the ACP activity in the L1 and L2 groups initially rose and then fell, with the L1 group decreasing by −4.44 ± 1.44 King unit/gprot at 36 h, while the L2 group increased by 18.82 ± 4.44 King unit/gprot at 24 h (Figure 2g). At 24 and 36 h, the ACP activity in the L2 group was significantly higher than in the L0 and L1 groups (*p* < 0.05, Figure 2h).

### 3.3. The Effects on Serum Metabolic Indicators

**Figure 3:**
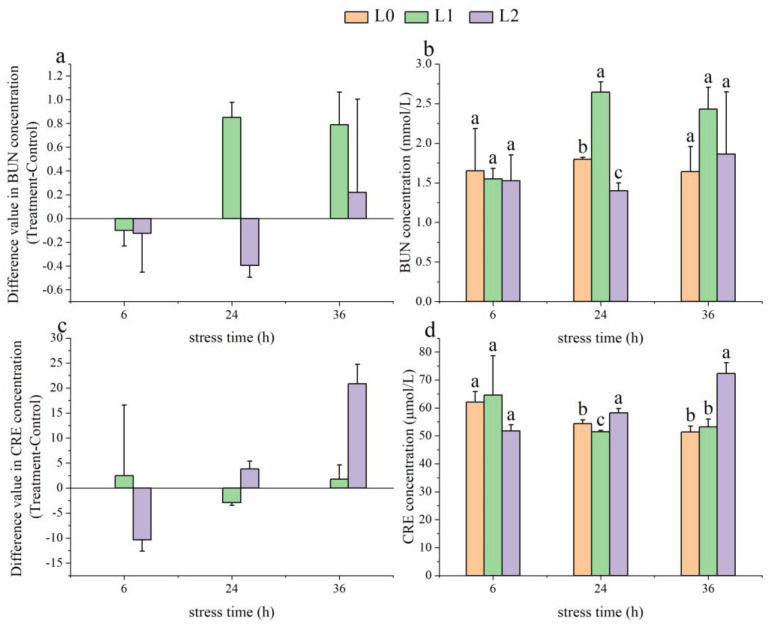
Effects of acute ammonia nitrogen stress on metabolic indices in the serum of juvenile yellowfin tuna (*Thunnus albacares*) (n = 9). (**a**) Difference value in blood urea nitrogen (BUN) concentration (treatment − control), (**b**) BUN concentration in treatment and control groups, (**c**) difference value in creatinine (CRE) concentration (treatment − control), and (**d**) CRE concentration in treatment and control groups. The difference value = treatment value − control mean value. Different letters indicate statistically significant differences (*p* < 0.05) between ammonia nitrogen concentrations at the same time point.

As time progressed, the BUN levels in the L1 group showed an initial increase followed by a decrease, rising by 0.85 ± 0.13 mmol/L at 24 h. In contrast, the BUN levels in the L2 group initially decreased and then increased, dropping by −0.40 ± 0.10 mmol/L at 24 h (Figure 3a). At 24 h, the BUN levels in the L1 group were significantly higher than those in the L0 group (*p* < 0.05), while the BUN levels in the L2 group were significantly lower than those in the L0 group (*p* < 0.05) (Figure 3b).

With increasing time, the CRE levels in the L1 group exhibited a trend of initially decreasing and then rising, decreasing by −2.92 ± 0.51 μmol/L at 24 h. The CRE levels in the L2 group showed an upward trend, increasing by 20.86 ± 3.87 μmol/L at 36 h (Figure 3c). At 36 h, the CRE levels in the L2 group were significantly higher than those in the L0 and L1 groups (*p* < 0.05) (Figure 3d).

### 3.4. The Effects on Spleen Antioxidant Gene Expression

**Figure 4:**
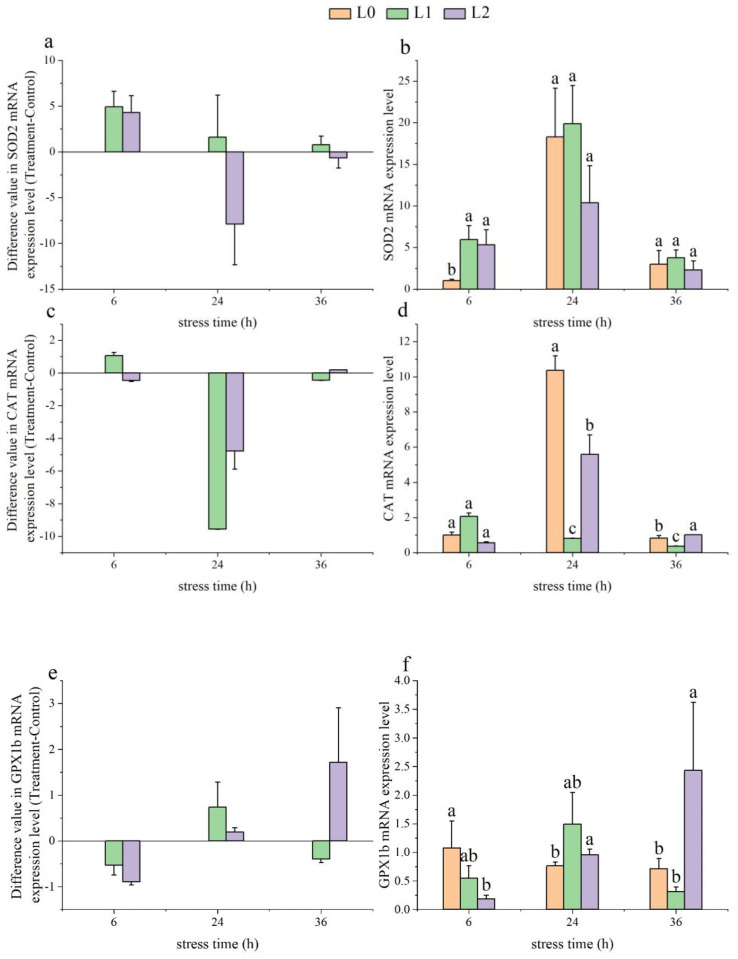
Effects of acute ammonia nitrogen stress on the expression of antioxidant genes in the spleen of juvenile yellowfin tuna (*Thunnus albacares*) (n = 9). (**a**) Difference value in superoxide dismutase 2 (*SOD2*) mRNA expression level (treatment − control), (**b**) *SOD2* mRNA expression level in treatment and control groups, (**c**) difference value in catalase (*CAT*) mRNA expression level (treatment − control), (**d**) *CAT* mRNA expression level in treatment and control groups, (**e**) difference value in glutathione peroxidase 1b (*GPX1b*) mRNA expression level (treatment − control), and (**f**) *GPX1b* mRNA expression level in treatment and control groups. The difference value = treatment value − control mean value. Different letters indicate statistically significant differences (*p* < 0.05) between ammonia concentrations at the same time point.

As time went on, the gene expression levels of *SOD2* in the L1 group showed a downward trend, while those in the L2 group initially decreased and then increased (Figure 4a). At 6 h, the *SOD2* gene expression levels in both the L1 and L2 groups were significantly higher than those in the L0 group (*p* < 0.05) (Figure 4b).

With increasing time, the gene expression levels of *CAT* in both the L1 and L2 groups exhibited an initial decrease followed by an increase (Figure 4c). At 24 h, the *CAT* gene expression levels in the L1 and L2 groups were significantly lower than those in the L0 group (*p* < 0.05). At 36 h, the *CAT* gene expression levels in the L2 group were significantly higher than in the L0 group (*p* < 0.05), while those in the L1 group were significantly lower than in the L0 group (*p* < 0.05) (Figure 4d).

As time progressed, the *GPX1b* levels in the L1 group initially rose and then fell, while the *GPX1b* levels in the L2 group showed a continuous increase (Figure 4e). The *GPX1b* gene expression levels in the L1 group showed no significant differences compared to the L0 group from 6 to 36 h (*p* > 0.05). However, the *GPX1b* gene expression levels in the L2 group were significantly lower than the L0 group at 6 h (*p* < 0.05) and significantly higher at 24 and 36 h (*p* < 0.05) (Figure 4f).

### 3.5. The Effects on Spleen Immune Gene Expression

**Figure 5:**
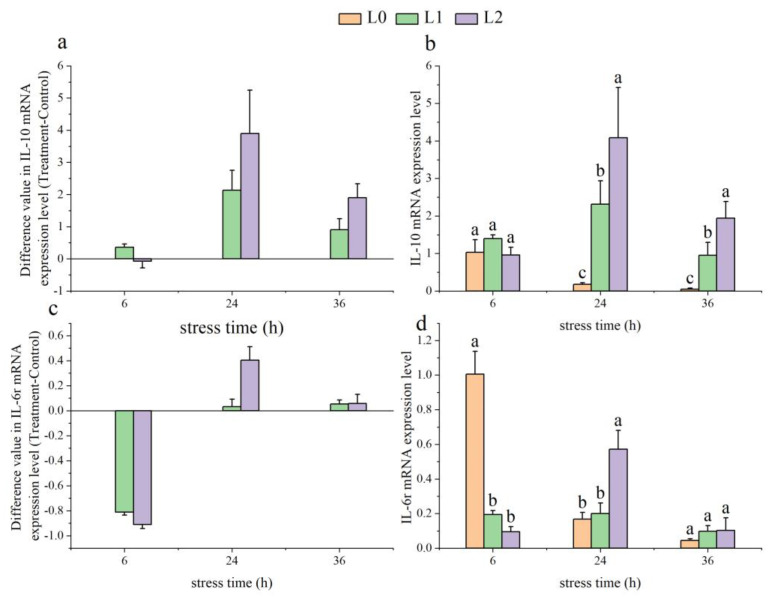

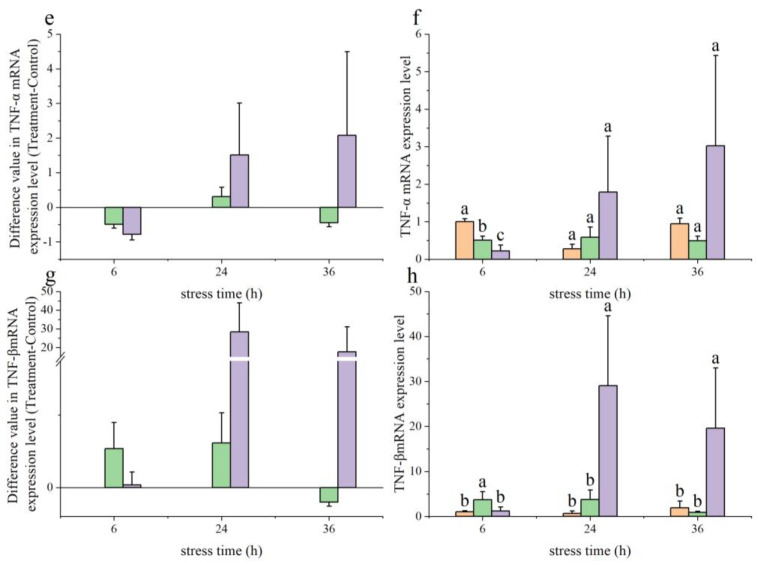
Effects of acute ammonia nitrogen stress on the expression of immunological genes in the spleen of juvenile yellowfin tuna (*Thunnus albacares*) (n = 9). (**a**) Difference value in interleukin 10 receptor (*IL-10*) mRNA expression level (treatment − control), (**b**) *IL-10* mRNA expression level in treatment and control groups, (**c**) difference value in interleukin 6r (*IL-6r*) mRNA expression level (treatment − control), (**d**) *IL-6r* mRNA expression level in treatment and control groups, (**e**) difference value in tumor necrosis factor a (*TNF-α*) mRNA expression level (treatment − control), (**f**) *TNF-α* mRNA expression level in treatment and control groups, (**g**) difference value in tumor necrosis factor a (*TNF-β*) mRNA expression level (treatment − control), and (**h**) *TNF-β* mRNA expression level in treatment and control groups. The difference value = treatment value − control mean value. Different letters indicate statistically significant differences (*p* < 0.05) between ammonia concentrations at the same time point.

As time increased, the gene expression levels of *IL-10* in the L1 and L2 groups initially rose and then fell (Figure 5a). At 24 and 36 h, the *IL-10* gene expression levels in both the L1 and L2 groups were significantly higher than those in the L0 group (*p* < 0.05) (Figure 5b).

With time, the *IL-6r* levels in the L1 group increased from 6 to 24 h, while the *IL-6r* levels in the L2 group showed an initial rise followed by a decline (Figure 5c). At 6 h, the *IL-6r* gene expression levels in both the L1 and L2 groups were significantly lower than in the L0 group (*p* < 0.05), and at 36 h there were no significant differences among the three groups (*p* > 0.05) (Figure 5d).

As time progressed, the *TNF-α* gene expression levels in the L1 group initially rose and then fell, while those in the L2 group exhibited a continuous increase (Figure 5e). At 6 h, the *TNF-α* levels in both the L1 and L2 groups were significantly lower than in the L0 group (*p* < 0.05) (Figure 5f).

With increasing time, the *TNF-β* gene expression levels in the L1 and L2 groups showed an initial rise followed by a decrease (Figure 5g). At 24 and 36 h, the *TNF-β* gene expression levels in the L2 group were significantly higher than in the L0 and L1 groups (*p* < 0.05) (Figure 5h).

### 3.6. The Effects on Spleen Apoptosis Factor Expression

**Figure 6:**
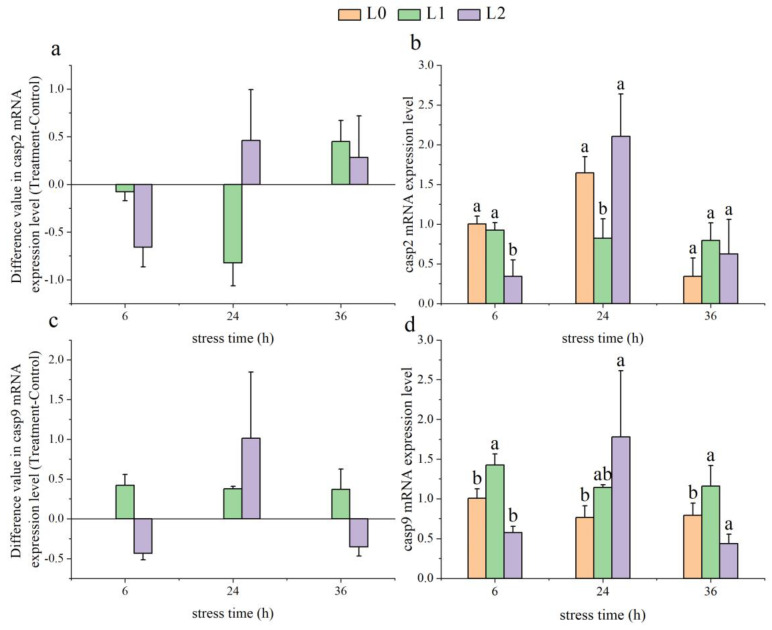
Effect of acute ammonia nitrogen stress on the expression of apoptotic factors in the spleen of juvenile yellowfin tuna (*Thunnus albacares*) (n = 9). (**a**) Difference value in caspase 2 (*casp2*) mRNA expression level (treatment − control), (**b**) casp2 mRNA expression level in treatment and control groups, (**c**) difference value in caspase 9 (*casp9*) mRNA expression level (treatment − control), and (**d**) *casp9* mRNA expression level in treatment and control groups. The difference value = treatment value − control mean value. Different letters indicate statistically significant differences (*p* < 0.05) between ammonia concentrations at the same time point.

As time progressed, the gene expression levels of *casp2* in the L1 group initially decreased and then increased, while those in the L2 group showed an initial rise followed by a decline (Figure 6a). At 6 h, the *casp2* gene expression levels in the L2 group were significantly lower than those in the L0 group (*p* < 0.05), and at 24 h, the *casp2* levels in the L1 group were significantly lower than in the L0 group (*p* < 0.05) (Figure 6b).

With increasing time, the gene expression levels of *casp9* in the L1 group showed no significant changes, while those in the L2 group exhibited an initial rise followed by a decline (Figure 6c). At 6 and 36 h, the *casp9* gene expression levels in the L1 group were significantly higher than those in the L0 group (*p* < 0.05), whereas the *casp9* levels in the L2 group were significantly lower than in the L0 group at both time points (*p* < 0.05, Figure 6d).

### 3.7. Summary Tables of Serum Biochemical Indicators and Spleen Gene Expression

A summary of the serum biochemical indicators is shown in Table 2. A summary of the spleen gene expression is shown in Table 3.

## 4. Discussion

### 4.1. Effects on Serum Biochemical Indicators

#### 4.1.1. Effects on Serum Lipid Metabolism

TG, T-CHO, LDL-C, and HDL-C are lipid substances within the body that play crucial roles in material transport and metabolism [14]. Research indicates that under low-salinity conditions, the activity of TG in juvenile Senegalese sole (*Solea senegalensis Kaup*) decreases [48]. Additionally, during the stress period, the serum levels of TG and T-CHO in Persian sturgeon (*Acipenser persicus*) showed a significant decrease at time zero compared to non-stressed conditions [49]. In this experiment, the serum levels of HDL-C and TG in the L1 group of juvenile yellowfin tuna initially decreased and then increased. This may be due to the juveniles enhancing their lipid metabolism capacity to cope with the effects of ammonia nitrogen in the early stages. As the duration of stress extended, they gradually adapted to the ammonia nitrogen environment, leading to a decline in lipid metabolism capacity. In the L2 group, LDL-C and HDL-C levels initially rose and then fell over time, while TG levels continued to rise. This may indicate that the 10 mg/L concentration of ammonia nitrogen disrupted the juvenile yellowfin tuna’s ability to handle ammonia nitrogen. The observed changes in metabolic biochemical indicators in the serum of juvenile yellowfin tuna may be attributed to the inhibitory effects of NH_3_-N. These alterations in lipid metabolites highlight the physiological adaptations and metabolic regulation of juvenile yellowfin tuna under stress. Understanding these responses is crucial for optimizing aquaculture practices and improving environmental conditions.

#### 4.1.2. Effects on Serum Immunobiochemical Indicators

C3 and C4 are essential components involved in regulating the fish immune system [50]. ACP and AKP are significant components of lysosomal enzymes in fish and play a crucial role in immune responses [51]. Research indicates that elevated levels of NH_3_-N can reduce the activity of C3 and C4 in the serum of juvenile turbot (*Scophthalmus maximus*) [52]. In this experiment, the serum levels of C3 and AKP activity in the L1 group initially increased and then decreased. In the L2 group, C3 levels and AKP activity showed an upward trend, while C4 levels and ACP activity demonstrated an initial increase followed by a decrease. This may indicate that juvenile yellowfin tuna exhibit enhanced immune system and metabolic regulation in the early stages of stress. However, as the stress persists, this response may shift to impaired immune and metabolic functions, suggesting that the fish may be unable to effectively cope with prolonged stress. The minimal effect of low NH_3_-N concentrations on immune substance activity contrasts with the increased activity observed at high NH_3_-N concentrations. These changes highlight the adaptive response of juvenile yellowfin tuna to varying levels of NH_3_-N, providing valuable insights into their immune regulatory mechanisms under environmental stress.

#### 4.1.3. Effects on Serum Metabolic Biochemical Indicators

BUN and CRE levels inversely reflect the glomerular filtration rate of the kidneys, with elevated levels indicating impaired kidney function and reduced filtration capacity [17,18]. In this experiment, the serum CRE levels in the L1 group showed a trend of initially decreasing and then increasing. This may indicate that the renal function of juvenile yellowfin tuna was suppressed under initial stress but recovered with adaptation, leading to increased CRE excretion. In the L2 group, the serum BUN levels also exhibited a trend of initially decreasing and then increasing, while the CRE levels showed an upward trend. This suggests that the juvenile yellowfin tuna may have initially responded to stress by slowing down their metabolism or reducing excretion, but subsequently, adaptation might have led to a restoration of the metabolic rate or an attempt by the kidneys to increase excretion. At the same time, the continuous rise in CRE levels indicates that renal function may be impaired, leading to ineffective CRE excretion. Ammonia nitrogen can harm the kidney health of juvenile yellowfin tuna, reducing glomerular filtration function. To protect the kidney health of these young fish, it is essential to regularly monitor ammonia nitrogen levels in the water and make timely adjustments.

### 4.2. Effects on Related Spleen Gene Expression

#### 4.2.1. Effects on Spleen Antioxidant Gene Expression

The spleen, as the largest secondary lymphoid organ in fish, plays a crucial role in blood filtration and preparation. It also serves as a key indicator of immune function, making it essential for assessing the immune status of fish [53,54]. Fish chronically exposed to NH_3_-N environments face the threat of oxidative stress, which can alter the expression patterns of antioxidant genes [55]. Studies have shown that under NH_3_-N stress conditions, antioxidant enzyme genes expression levels in the gills of golden pompano (*Trachinotus ovatus*), such as *SOD*, *CAT,* and glutathione peroxidase (*GPX*), increase [56]. In this experiment, the expression of *SOD2* in the spleen of the L1 group showed a decreasing trend, while the expression of the *GPX1b* gene exhibited a pattern of first increasing and then decreasing. This suggests that juvenile yellowfin tuna initially attempted to cope with oxidative stress by increasing *GPX1b* expression, but as stress continued, their antioxidant defense system may have been compromised by sustained ammonia levels, preventing both *SOD2* and *GPX1b* from maintaining effective levels. In the L2 group, *SOD2* expression in the spleen initially decreased and then increased, while the *GPX1b* expression continued to rise. This indicates that juvenile yellowfin tuna may have experienced a decline in antioxidant defense capacity due to the initial stress responses but subsequently actively responded to ongoing oxidative pressure by upregulating the expression of *SOD2* and *GPX1b*, aiming to restore and enhance their antioxidant capacity. The above findings reveal the stress response mechanisms in the spleen of juvenile yellowfin tuna under varying NH_3_-N concentrations. This provides scientific theoretical support for the effective management of NH_3_-N in aquaculture, contributing to the improvement of juvenile yellowfin tuna health. Consequently, this research enhances the overall efficiency and quality of aquaculture products.

#### 4.2.2. Effects on Related Spleen Immune Gene Expression

The spleen, which contains lymphocytes, functions as a key immune organ that responds to antigens entering the bloodstream [53]. In response to NH_3_-N induction, the pufferfish (*Takifugu obscurus*) exhibits upregulation in the transcription of inflammatory cytokines such as *TNF-α* and IL-6 [52]. In this experiment, the expression of *IL-10* and *TNF-α* in the L1 group, as well as *IL-10*, *IL-6r*, and *TNF-β* in the L2 group, showed a trend of initially increasing and then decreasing. This may reflect the fish’s response to stress by enhancing immune reactions initially, followed by self-regulation to avoid excessive inflammation. Meanwhile, the continuous rise in *IL-6r* expression in the L1 group and *TNF-α* expression in the L2 group may indicate that the fish require sustained immune regulation to cope with ongoing environmental stress. These findings highlight the importance of maintaining appropriate NH_3_-N levels in aquaculture environments to ensure that the fish’s immune system can effectively manage environmental stresses.

#### 4.2.3. Effects on Spleen Apoptosis Factor Expression

When stress responses become severe enough to disrupt the immune system, cells may experience significant damage and trigger the expression of apoptosis factors. Caspases are crucial indicators for detecting apoptosis in fish [57]. Under low-temperature stress conditions, the liver cell apoptosis mechanisms in orange-spotted grouper and pufferfish are activated, leading to the activation of caspase family proteases [58,59]. Under ammonia stress, the trend of *casp2* and *casp9* gene expression in the fish spleen showed an initial increase followed by a decrease, revealing the early activation of the apoptotic process followed by subsequent self-regulation. This suggests that fish, in response to environmental stress, initiate apoptotic programs to clear damaged cells, and later employ regulatory mechanisms to reduce excessive apoptosis, thereby protecting the normal function of tissues and organs. This self-regulation may help fish to survive under ammonia stress, but sustained high ammonia levels could pose a threat to their health, highlighting the need for measures to alleviate ammonia stress and protect fish health.

Under low concentrations of NH_3_-N, the expression levels of antioxidant genes, immune enzymes, and apoptotic factors in the spleen of juvenile yellowfin tuna are relatively low. However, when NH_3_-N concentrations increase, the expression of pro-inflammatory cytokines and apoptotic factors significantly rises. Therefore, to protect the spleen of juvenile yellowfin tuna from damage, it is essential to strictly control the concentration of ammonia nitrogen in the water.

## 5. Conclusions

This experiment investigated the effects of three NH_3_-N concentrations (0, 5, and 10 mg/L) on serum biochemical indicators and gene expression in the spleen of juvenile yellowfin tuna at various time points (6, 24, and 36 h). The results indicated that when NH_3_-N levels ranged from 0 to 5 mg/L and the stress duration varied between 6 and 36 h, the impact on serum biochemical indicators and gene expression in the spleen was minimal. However, at ammonia nitrogen concentrations of 5–10 mg/L and stress durations of 6 to 24 h, the levels of metabolic-related serum indicators (TG), immune-related indicators (C3, AKP), and glomerular permeability (CRE) showed an increasing trend. Based on these findings, it is recommended that in the artificial cultivation of juvenile yellowfin tuna, measures be taken to mitigate the adverse effects of NH_3_-N. Specifically, when NH_3_-N concentrations are between 0 and 5 mg/L, the stress duration should not exceed 36 h. For concentrations between 5 and 10 mg/L, the stress duration should be limited to 24 h or less. Implementing these management strategies will help to create a controlled aquaculture environment, reduce the risk of disease and environmental pollution, and support the conservation and sustainable utilization of wild yellowfin tuna.

## Figures and Tables

**Table 1:** RT-PCR primer sequence.

Gene		Full Name of the Gene	Primer Sequences	Amolification Size
*SOD2*	F	superoxide dismutase 2	CGGGACTTTGGTTCCTTCCA	128
*SOD2*	R		GCACAAGCAGCGATACGAAG	
*CAT*	F	catalase	CAGGCAACAACACCCCCA	122
*CAT*	R		CCAGAAGTCCCACACCAT	
*GPX1b*	F	glutathione peroxidase 1b	GACCACCAGGGATTACAC	150
*GPX1b*	R		GGACGGACATACTTCAGA	
*IL-6r*	F	interleukin 6 receptor	TTGTCAGTCATTTTGGCT	132
*IL-6r*	R		CTCTGGAGATGTTGGGGT	
*IL-10*	F	interleukin 10	CAGCAAGATACCAACAAG	190
*IL-10*	R		CGACAAGAGAACCAGGAC	
*TNF-α*	F	tumor necrosis factor a	ACAGCCAGGCATCTTTCC	116
*TNF-α*	R		GGCGTCACCGTTCCCATA	
*TNF-β*	F	tumor necrosis factor b	GGGACCCTCCTCATCATC	194
*TNF-β*	R		CTTCGCAAAACCCTTCTA	
*casp2*	F	caspase 2	CAACACTCCTGTGCTCCC	166
*casp2*	R		ATCCACCTCACCTCCCTT	
*casp9*	F	caspase 9	GGTGCCGTGTATGGTGTG	116
*casp9*	R		GCCTGGATGAAGAAGAGT	
*β-actin*	F	β-actin	CGCCCTCGTTGTTGAC	170
*β-actin*	R		CCCTTTTGCTCTGTGCC	

**Table 2:** Serum biochemical indicators summary.

Enzyme Activity	L1	L2
LDL-C	↓↓	↑↓
HDL-C	↑↓	↑↓
T-CHO	↓↓	↓↓
TG	↑↓	↑↑
C3	↑↓	↑↑
C4	-↓	↑↓
AKP	↓-	↑↑
ACP	↑↓	↑↓
BUN	↑-	↓↑
CRE	↓↑	↑↑

Note. The first symbol indicates the trend of the change in enzyme activity over time for the periods of 6–24 h, and the second symbol represents the trend from 24–48 h. ↑ indicates an increasing trend, ↓ indicates a decreasing trend, and - indicates a stable trend.

**Table 3:** Spleen gene expression summary.

Gene	L1	L2
*SOD2*	↓↓	↓↑
*CAT*	↓↑	↓↑
*GPX1b*	↑↓	↑↑
*IL-10*	↑↓	↑↓
*IL-6r*	↑↑	↑↓
*TNF-α*	↑↓	↑↑
*TNF-β*	-↓	↑↓
*casp2*	↓↑	↑↓
*casp9*	--	↑↓

Note. The first symbol indicates the trend of the change in gene mRNA expression over time for the periods of 6–24 h, and the second symbol represents the trend from 24–48 h. ↑ indicates an increasing trend, ↓ indicates a decreasing trend, and - indicates a stable trend.

## Data Availability

The original contributions presented in the study are included in the article. Further inquiries can be directed to the corresponding authors.
